# Antiretroviral Treatment Knowledge and Stigma—Implications for Programs and HIV Treatment Interventions in Rural Tanzanian Populations

**DOI:** 10.1371/journal.pone.0053993

**Published:** 2013-01-16

**Authors:** Abela Mpobela Agnarson, Francis Levira, Honorati Masanja, Anna Mia Ekström, Anna Thorson

**Affiliations:** 1 Department of Public Health Sciences, Division of Global Health (IHCAR), Karolinska Institutet, Stockholm, Sweden; 2 Ifakara Health Institute, Dar es Salaam, Tanzania; University of Washington, United States of America

## Abstract

**Objective:**

To analyse antiretroviral treatment (ART) knowledge and HIV- and ART-related stigma among the adult population in a rural Tanzanian community.

**Design:**

Population-based cross-sectional survey of 694 adults (15–49 years of age).

**Methods:**

Latent class analysis (LCA) categorized respondents' levels of ART knowledge and of ART-related stigma. Multinomial logistic regression assessed the association between the levels of ART knowledge and HIV- and ART-related stigma, while controlling for the effects of age, gender, education, marital status and occupation.

**Results:**

More than one-third of men and women in the study reported that they had never heard of ART. Among those who had heard of ART, 24% were east informed about ART, 8% moderately informed, and 68% highly informed. Regarding ART-related stigma, 28% were least stigmatizing, 41% moderate*l*y stigmatiz*i*ng, and 31% highly stigmatizing toward persons taking ART. Respondents that had at least primary education were more likely to have high levels of knowledge about ART (OR 3.09, 95% CI 1.61–5.94). Participants highly informed about ART held less HIV- and ART-related stigma towards ART patients (OR 0.26, 95% CI 0.09–0.74).

**Conclusion:**

The lack of ART knowledge is broad, and there is a strong association between ART knowledge and individual education level. These are relevant findings for both HIV prevention and HIV treatment program interventions that address ART-related stigma across the entire spectrum of the community.

## Introduction

The “Universal access initiative” to scale up HIV prevention, care and treatment coverage in resource-poor settings was also associated with hopes to lower stigma and discrimination against people living with HIV (PLHIV). However, recent studies have revealed that HIV-related stigma still harms uptake and optimal utilization of HIV services [1], [2], regardless of increased availability of antiretroviral therapy (ART) [3], [4]. Previous qualitative work has also shown that increased access to ART may increase stigmatizing attitudes towards PLHIV on ART [5], [6]. Negative attitudes towards ART patients may partly explain the current slow enrollment rate into HIV treatment in many rural populations living in high-burdened HIV contexts.

HIV prevalence in Tanzania stabilized between 6.5% and 7% in since 2008, and adult ART coverage was at 42% in 2010 compared to the 16% in 2008 [7]. This study builds on an earlier qualitative study conducted in the same population in Tanzania [5]. The study, conducted in 2008, explored challenges to ART scale-up among health workers, ART patients and community members. That specific study disclosed adverse beliefs and perceptions about ART and stigma against ART patients (hereafter referred to as ART-related stigma). Community members thought of ART as a means to extend life only for a brief period of time and employed the expression; *“marehemu mtarajiwa”-“dead to be”* to describe patients on ART. Community members had, generally, observed individuals starting ART and then dying quickly thereafter, an experience that had probably led to the belief that ART provides only the person with time to contemplate his or her funeral.

In the present study, a distinction is made between HIV-related stigma and ART-related stigma. HIV is the common denominator in both HIV-related stigma and ART-related stigma. HIV-related stigma may act at a social and individual level, as PLHIV may experience labeling, stereotyping, discrimination, separation and loss of status [8]. These are all theorized concepts that to lead PLHIV to be less open about their HIV status and to inhibit them from seeking preventative, testing or treatment services [9]. Previous studies suggest that ART-related stigma is linked to the community's understanding that an HIV-infected person, after starting ART and achieving symptomatic improvement, is able to hide his or her HIV infection and intentionally transmit HIV to others [5], [6], [10]. Hence, we postulate that ART-related stigma is an extension of the already-existing HIV-related stigma against HIV-positive people, grounded in the fear of becoming infected with HIV from that person.

Based on our previous qualitative findings, our hypothesis was that access to ART in the community was accompanied by stigmatizing attitudes towards ART patients in the community. The aim of the present study was to analyse ART knowledge and HIV- and ART-related stigma in the general population aware of ART in a rural Tanzanian community to inform development of a targeted intervention.

## Methods

### Ethics statement

The national ethics committee of Tanzania (Medical Research Coordinating Committee) and the Tanzania Commission for Science and Technology (COSTECH) approved this study, (NIMR/HQ/R.8a/VolIX/609). The study was conducted as a module within a continuing routine data collection process within the Rufiji Demographic Surveillance System's (RDSS). Informed consent process in a RDSS has previously been described, however, written informed consent was received from each study participant [11], [12].

### Study Setting and the Rufiji Demographic surveillance system

Rufiji district is located in the rural coastal area in the Pwani Region, located 180 kilometres south of Dar es Salaam, and has a population of approximately 200,000 [11]. In 2008, the regional HIV prevalence was 5.6% [13]. In March 2005, the first ART treatments were initiated and by December 2010 around 1,402 adults aged 15–49 years in the district had been initiated on ART.

#### Rufiji Demographic Surveillance System

The study data was collected from the Rufiji Demographic Surveillance System (RDSS) site in Rufiji District, managed by the Ifakara Health Institute (IHI) on behalf of the Ministry of Health and Social Welfare. The RDSS monitors a total population of 93,000 people in 18,000 households in 32 villages. All residents are monitored regularly, every 3–4 months, through household surveys performed by fieldworkers interviewing household members about all births, deaths, in-migrations, out-migrations, and pregnancies.

### Survey design

#### Sample

Interviews were performed by RDSS full-time enumerators deployed in the villages from January to March 2009. A population-based sub-sample of 1000 adults, 15–49 years of age, was drawn from the RDSS. We calculated the need for a sample of 800 based on a power of 80% to account for dropouts. We then adjusted the sample by adding about 20% to cater for dropouts. The RDSS area is composed of 32 villages that were stratified by gender and age (15–24 years, 25–34 years & 35–49 years). An equal number of individuals were selected from each stratum to ensure a representative sample. A total of 166 were out-of-reach, 45 declined to participate, and 95 had incomplete survey information, yielding a total of 694 respondents (69% of the originally drawn sample). Socio-demographic comparison between the respondents and non-respondents did not show any significant discrepancies in age, educational level, or occupation between the two groups (Table S1). However, males were less likely to agree to be interviewed as compared to females (OR 0.67, 95% CI 0.51–0.88). Individuals cohabiting or married (OR 1.65, 95% CI 1.23–2.20) were more likely to respond in the survey. We did not evaluate the HIV status of respondents for this study.

#### Data collection and generating the items

The questionnaire was divided into four parts covering the themes described below. Participants responded to items as ‘Yes’, ‘No’, or ‘Don't know’. RDSS enumerators and the research team reviewed these items during training and piloting.

Items on ART community awareness, ART knowledge and ART-related stigma were derived from two categories in a previous qualitative study [5]: (i) community knowledge, attitudes and acceptance of ART and (ii) ART-related stigma in the community and sexual risk taking among ART patients.

ART community awareness: Two items pertained to the source of ART information. Respondents were asked if they had ever heard of ART, and where they had heard of ART.

ART knowledge: Five items covered ART knowledge: ART is obtained free of charge from hospital, an HIV-infected pregnant woman can be treated with ART, ART can prolong life, ART is life-long treatment and ART is used only when someone is very ill.

ART-related stigma: These items focused on attitudes towards ART patients and ART: ART patients are a threat to society, are intentionally transmitting HIV, ART patients look healthy after taking ARVs and transmit HIV to others, ARVs increases sexual drive for ART patients, ART patients are *“dead to be”* and ART patients will die soon.

HIV-related stigma: The HIV-related stigma items included in the study originated from stigma concepts of labeling, stereotyping, separation and discrimination presented by Link and Phelan [14]. Moreover, these items have been used in other HIV-related stigma studies in similar sub-Saharan Africa settings [15], [16], [17]. Five items described negative beliefs about PLHIV: PLHIV are a threat to society, PLHIV are dangerous to me, PLHIV should be isolated from others, PLHIV are repellant, and I do not want to be friends with PLHIV.

### Statistical analysis

#### Data management and descriptive statistics

We carried out standard data cleaning that involved identifying outliers and incomplete records. We then merged the data set with the demographic surveillance data system to link it with demographic characteristics such as marital status, education, and occupation. We executed descriptive analyses using Stata software, version 10 (STATA Corporation, College Station, TX, 2005), and chi-square tests were used to measure the significance of bivariate associations.

#### Latent class analysis

We used LCA because it identifies homogeneous classes within a heterogeneous population based on similarity of responses to measured variables (item response), and covariates that differentiated membership across classes [18], [19]. Several different academics have applied LCA to assess HIV knowledge, quality of life, psychosocial distress and risk behavior among diverse society groups and domains [20], [21], [22].

Initially, an optimum number of classes was estimated by fitting a series of LCA-restricted models with two to five classes using Akaike information criterion (AIC) and Bayesian information criteria (BIC) [23]. The adjusted BIC was used to assess the model of optimal fit (Table S4). The entropy parameter measures how well the model predicted class memberships; the higher values indicated a clearer class separation.

After obtaining an optimum number of classes on ART knowledge, ART-related stigma and HIV-related stigma, a latent class multinomial logistic regression model was fitted (one for each construct), which included the demographic covariates: age, gender, education, age, marital status and occupation. The use of backward model selection eliminated non-significant covariates in each analysis. Odds ratios (OR) and 95% confidence intervals (CI) are presented for each cross-classification. This study used SAS software (Version 9.2 Cary, NC: SAS Institute Inc) and the PROC LCA method to conduct the LCA analysis using a stepwise approach [18].

## Results

### Descriptive analyses of the rural men and women independent of HIV and ART status

#### ART community awareness

An aggregate of 694 individuals (306 men, 388 women) responded to the survey. However, a large number, 34% of respondents (105 men, 134 women), with a mean age of 29.1 (SD±10.2), had never heard about ART (Table S2). This sub-group was only included in the descriptive analysis, resulting in 455 participants that constituted the LCA analysis.

There were gender differences in how knowledge about ART had been obtained (Table S3). Significantly lower proportion of women than men had heard about ART from TV or radio (49% vs. 62%, p<0.01) and from posters (15% vs. 24%, p<0.05). A significantly greater proportion of women than men had received information from health workers (48% vs. 29%, p<0.001).

#### ART knowledge

One-third of 455 respondents who had heard about ART were uninformed that ART could be accessed for free from the hospital (Table S3). Moreover, respondents showed inadequate knowledge on treatment and prevention for HIV infected pregnant women: only 34% knew that HIV-infected pregnant woman could be on ART. Nearly 20% thought that ART does not prolong life, and 47% did not know that antiretrovirals (ARVs) should be used throughout life. Few (8%) thought that ARVs should be used only when PLHIV are very ill.

#### ART-related stigma

Many participants believed that ART patients are a threat to society (57%) and that they intentionally transmit HIV to others (45%, Table S3). In addition, 38% of respondents believed that ART patients look healthy after taking ARVs and transmit HIV to others.

There was a significant difference between the proportion of men and women endorsing this item (43% vs. 34%, p<0.05). Forty-six percent of respondents thought that ARVs increase ART patients' sex drive; a significant gender difference also existed for this item (45% of men vs. 47% of women, p<0.05). Also of note, 38% of respondents assumed that ART patients are *“dead to be”* (a derogatory expression frequently used in the previous study by participants to delineate a person on ART [5]), while only 10% conjectured that PLHIV on ART would die soon. The difference is likely since *“dead to be”* is a more fluid, imprecise concept, where according to the qualitative results, ART patients are considered to live with a constant threat of dying, whereas “dying soon” is being interpreted as actually being on the verge of dying within the next few years.

#### HIV-related stigma

Notably, about 63% of the respondents stated that PLHIV are a threat to society, 56% considered PLHIV to be dangerous to respondents and 55% felt that PLHIV should be isolated (Table S3). Moreover, 12% considered PLHIV disgusting, and 11% did not want to be friends with a person infected with HIV.

### Latent class analysis results

We examined latent classes in the three main constructs (*ART knowledge, ART-related stigma* and *HIV-related stigma*) separately and in a combined LCA model. Table S4 shows the latent class analysis model fitted for the three main constructs. Table S5 shows the latent class entities for ART knowledge and ART-related stigma. Table S6 contains the latent class multinomial logistic regression model on association between the main constructs.

#### ART knowledge

LCA classified three groups of respondents distinctive ART knowledge levels based on response patterns: 24% are in the least informed, 8% in the moderately informed group, and 68% in the group of persons that is well informed on ART knowledge (Table S5). In the least informed class, the majority demonstrated good knowledge about where ART could be obtained (81%) and low knowledge on the other items. In the moderately informed class, 99% of respondents correctly identified the hospital as the place where to get ART, while 54% knew of prevention of mother-to child transmission. The highly informed group consisted of respondents that scored highly on all above-mentioned indicators, except on the item stating that ART should be used only when the person is very ill.

The effect of formal education on ART knowledge was estimated by fitting Model 1 (Table S6), where the response variable was the three classes of ART knowledge and the determinant of interest was a primary school level of education or higher. According to the multinomial logistic regression, respondents that had no formal education were the least informed about ART and expressed the highest HIV-related stigma. Respondents that had at least a primary education level were three times more likely to be highly knowledgeable about ART (OR 3.09, 95% CI 1.61–5.94).

#### ART-related stigma

Among the identified LCA groups for ART-related stigma, 28% of respondents belonged to the least stigmatizing, 41% as moderately stigmatizing, and 31% as highly stigmatizing toward persons taking ART (Table S5). Notably, 94% of individuals in the highly stigmatizing group perceived ART patients as *“dead to be”*, and 99% of members in the same group believed that these patients would die soon. Furthermore, the results show that the term *“dead to be”* is frequently used across the three ART-related stigma classes.

Model 3 was fitted for the latent class response variable ART-related stigma (Table S6). In this model ART knowledge and HIV-related stigma are predictors while we adjusted for education level. Participants with high HIV-related stigma were more likely to express moderately (OR 42.15, 95% CI 43.32–57.6) and highly stigmatizing (OR 61.57, 95% CI 55.40–72.50) attitudes towards patients on ART as compared to those with low HIV-related stigma. Participants that were well informed about ART are less likely to express highly stigmatizing attitudes towards patients on ART (OR 0.26, 95% CI 0.09–0.74), than were respondents with the lowest level of ART knowledge. These findings suggest that ART knowledge is closely linked to ART-related stigma. There was no association between ART-related stigma and formal education, after adjustment for ART knowledge and HIV-related stigma (both highly correlated with education level).

#### HIV-related stigma

Two groups were defined: low HIV-related stigma (57% or respondents) and high levels of HIV-related stigma (43% of respondents).

Association between the binary latent class response variable (low and high HIV-related stigma) and ART knowledge was fitted controlling for formal education (Model 2, Table S6). Participants with at least primary education were less likely to show the highest level of stigmatizing attitudes (OR 0.58, 95% CI 0.35–0.95) compared to participants that had no formal education. Participants that were moderately and highly informed about ART were less likely to show high HIV-related stigma (OR 0.35, 95% CI 0.13–0.92; and OR 0.44, 95% CI 0.23–0.81, respectively) as compared to participants that were least informed about ART.

## Discussion

To our knowledge, this is the first population-based study about ART stigma in the general population in rural Sub-Saharan African. It is clear from the descriptive findings and latent class models that HIV-related stigma might still be an impediment to ART patient retention and linkage to care. In Tanzania, only 82% of ART patients are known to be on ART one year after starting on ART [24].

More than a third of men and women in the study had never heard of ART. This study suggests that hostility towards ART patients is strongly grounded in lack of adequate knowledge about ART. In addition, from a multisite study in Tanzania, patients on ART reported greater experience of HIV-related stigma compared to those individuals who had not started on ART [25]. Other studies found that being on ART was seen as an indication of severity of the illness, rather than as something that would slow disease progression and improve health. The community's fear of not being able to identify an HIV-infected person appeared to drive hostility against ART patients. The potentially hostile environment created towards ART patients may lead people to be more reluctant to start on ART, or may lead PLHIV on ART not to disclose their status, possibly continuing risky sexual activity, with the risk of reinfection or transmission, or leading to treatment non-adherence [26].

Lack of formal education and rural residence are highly correlated to lack of knowledge of HIV prevention and non-accepting attitudes towards those living with HIV [27], [28]. Most of women acquired information on ART from health workers; however, the link between source of ART information and level of ART-related stigma was not explored in this specific study. However, health literacy is necessary to be able to process and evaluate information provided in mass media or the information provided through medical services [29], [30].

To effectively achieve and optimize widespread ART coverage in rural Tanzania settings, we ardently encourage broadened educational and programmatic efforts through interpersonal communication at the community level. This is a suggestion that can create an impact through the use of village AIDS committees (VACs). VACs are already established in every Tanzanian village by the government as a strategy to enhance local authority in managing the burden of HIV at local level [31]. This is the context-adjusted channel to disseminate information about HIV treatment, as well as to carry out the work that challenges ART-related stigma towards ART patients in the community, provided that this group receives adequate training on the subject.

A high percentage of men and women in our study were unaware that HIV-infected pregnant women should use ART to prevent mother to child transmission. This lack of knowledge is very serious, because it reflects lost opportunities to save mothers' lives and to avert infections in children [32].

A lower percentage of men, as compared to women, used the health facility as a source of information about ART. The low reliance on the health system by men highlights the existing gender inequity in access to HIV care. Remarkable advancement has been made in resource-poor settings in expanding access to ART; however, patients have been disproportionately female [33]. Men are also more likely to start on the treatment at a late stage of disease [34] and to interrupt treatment [35].

To our knowledge, there are not any published quantitative studies specifically examining stigma towards PLHIV on ART in general population samples in sub-Saharan Africa. The present study relied on previous published formative qualitative research conducted in the same setting. Although the context is of importance to stigma construction, the broad literature on stigma towards PLHIV in low-income countries shows very similar results cross-culturally [36], [37]. The stigma items used were drawn from studies that have used validated stigma scales, thus enhancing the reliability and validity of the study.

About 69% of the targeted population responded to the survey. Potential sources of bias were minimized through running a drop-out analysis on gender, age, education and socio-economic status, and by comparing respondents to non-respondents to the survey. Participants' HIV status was not examined in the analysis and since the local HIV prevalence is estimated at 6%, only a small number of participants would know of their HIV-positive status, thus having minor impact on the key variables examined. The data are cross-sectional, limiting the ability to establish a causal direction for observed patterns of ART knowledge, HIV-related stigma and ART-related stigma; nevertheless, this was not in the scope of the study.

The LCA analysis enabled the categorization of individuals with similar beliefs on PLHIV, ART patients and knowledge of ART into groups, providing the possibility to efficiently explore the extent to which these beliefs were socio-demographically distributed. Like most multivariate analysis methods, LCA is eminently dependent on inputs included in the model and open to multiple interpretations. Dichotomizing of continuous variables is a common approach in LCA modeling that aids the interpretation and communication of the findings. It should be noted, that the dichotomization of the data might also introduce a loss of sensitivity.

Notwithstanding these limitations, we have established a significant relationship between education level, ART knowledge, HIV-related stigma, and negative perceptions towards ART patients. These findings are relevant for both HIV prevention and HIV treatment program interventions that address knowledge and beliefs about ART and ART patients among those lacking formal education and across the entire spectrum of the community.

## Supporting Information

Table S1 **Comparison of participants to non-participants who did not consent or provided incomplete information (third column from left).**
(DOC)

Table S2 Descriptive information about the 694 participants in the study. *Indicates a significant difference at p<0.05.
(DOC)

Table S3 **Characterizations of ART awareness, ART knowledge, ART-related knowledge and HIV-related stigma between men and women participating in the study.** The answers provided in this table are positive answers. *It describes 694 participants of the 1000 individuals targeted in the survey. **Total sample = 455 (all respondents with non-missing ID or gender data that have heard of ART). Sample sizes for individuals vary somewhat due to different response pattern on each item. ^§^At time of the study, ART was only accessible in two hospitals in the district.
(DOC)

Table S4 **Fit indices for the LCA of ART knowledge, HIV-related stigma and ART-related stigma.** Best fitting model is in bold. LCA = latent class analysis; AIC = Aikike Information Criterion; BIC = Bayesian Information Criterion; Adjusted BIC = Bayesian Information Criterion using sample size adjustment. *Number of latent classes restricted to education as the main grouping variable to make equal various conditional response probabilities across pairs of latent classes.
(DOC)

Table S5 **The latent class entities for ART knowledge and ART-related stigma.** *Estimated conditional probabilities and membership probabilities (proportions) for each class based on “Yes” responses to the items. Bold numbers denote the most recurrent answer within the class to each statement.
(DOC)

Table S6 **Multinomial logistic regression models on ART knowledge, HIV-related stigma and ART-related stigma latent groups.** Description on significant covariates at p<0.0001. The models were adjusted for; age, education, gender, marital status and occupation. Reference covariate and classes: *No formal education, ^†^Least informed, **Low HIV-related stigma and ^§^ Least stigmatizing. Adjusted odds ratio and 95% confidence intervals.
(DOC)
