# Supplementary material for: Antiretroviral Treatment Knowledge and Stigma—Implications for Programs and HIV Treatment Interventions in Rural Tanzanian Populations
Source: PLoS One. 2013 Jan 16;8(1):e53993. doi: 10.1371/journal.pone.0053993 (PMC3546967; doi:10.1371/journal.pone.0053993)
Supplement: Table S1 — Comparison of participants to non-participants who did not consent or provided incomplete information (third column from left). (DOC) [file pone.0053993.s001.doc]

**Table S1. Comparison of participants to non-participants who did not consent or provided incomplete information (third column from left)**

|  | Interviewed | Non- interviewed | Total |  |  |
| --- | --- | --- | --- | --- | --- |
|  | N=694 | N=306 | 1000 |  |  |
| Mean age, ±SD | 30.1, ±9.5 | 29.5, ±9.3 | 29.9, ±9.5 |  |  |
|  |  |  |  |  |  |
| **Age** | Frequency (%) | Frequency (%) | Frequency (%) | OR | 95% CI |
| 15-25 years | 229(33) | 102(33) | 331(33) |  |  |
| 25-34 years | 224(32) | 106(35) | 330(33) | 0.94 | 0.68-1.31 |
| 35-44 years | 186(27) | 70(23) | 256(26) | 1.18 | 0.83-1.70 |
| 45-49 years | 55(8) | 28(9) | 83(8) | 0.87 | 0.52-1.46 |
|  |  |  |  |  |  |
| **Gender** |  |  |  |  |  |
| Females | 388(73) | 141(27) | 529(53) |  |  |
| Males | 306(65) | 165(35) | 471(47) | 0.67 | 0.51-0.88 |
|  |  |  |  |  |  |
| **Education history** |  |  |  |  |  |
| No education | 191(70) | 83(30) | 274(27) |  |  |
| Formal education | 503(69) | 223(31) | 726(73) | 0.98 | 0.72-1.33 |
|  |  |  |  |  |  |
| **Marital status** |  |  |  |  |  |
| Never married | 232(65) | 126(35) | 358(36) |  |  |
| Married or cohabiting | 419(75) | 138(25) | 557(56) | 1.65 | 1.23-2.20 |
| Divorced | 37(70) | 16(30) | 53(5) | 1.26 | 0.67-2.35 |
| Widowed | 5(56) | 4(44) | 9(1) | 0.68 | 0.18-2.57 |
| Missing | 1(4) | 22(96) | 23(2) | 0.02 | 0.00-0.19 |
|  |  |  |  |  |  |
| **Occupation** |  |  |  |  |  |
| Student | 111(71) | 46(29) | 157(16) |  |  |
| Farmers | 385(74) | 133(26) | 518(52) | 1.20 | 0.81-1.78 |
| Employed *(government/private)* | 147(66) | 77(34) | 224(22) | 0.79 | 0.51-1.23 |
| Unemployed | 44(70) | 19(30) | 63(6) | 0.96 | 0.51-1.82 |
| Missing | 7(18) | 31(82) | 38(4) | 0.09 | 0.04-0.23 |
