# Supplementary material for: Antiretroviral Treatment Knowledge and Stigma—Implications for Programs and HIV Treatment Interventions in Rural Tanzanian Populations
Source: PLoS One. 2013 Jan 16;8(1):e53993. doi: 10.1371/journal.pone.0053993 (PMC3546967; doi:10.1371/journal.pone.0053993)
Supplement: Table S2 — Descriptive information about the 694 participants in the study. *Indicates a significant difference at p<0.05. (DOC) [file pone.0053993.s002.doc]

**Table S2. Descriptive information about the 694 participants in the study**

*Indicates a significant difference at p<0.05

|  | Men | Women | Total |
| --- | --- | --- | --- |
| *Participants who had never heard of ART not included in the LCA analysis* | N=105 | N=134 | N=239 |
| Mean age, ±SD | 28.0, ±10.8 | 30.0, ±9.7 | 29.1, ±10.2 |
| *Participants who had heard of ART included in the LCA analysis* | N=201 | N=254 | N=455 |
| Mean age, ±SD | 31.6, ±10.0 | 31.8, ±9.0 | 31.7, ±9.4 |
|  | Frequency (%) | Frequency (%) | Frequency (%) |
| **Age** |  |  |  |
| 15-24 years | 60(30.0) | 61(24.0) | 121(26.6) |
| 25-34 years | 63(31.3) | 92(36.0) | 155(34.1) |
| 35-44 years | 50(25.0) | 75(29.5) | 125(27.5) |
| >45 years | 28(14.0) | 26(10.5) | 54(12.0) |
| **Marital status** |  | | |
| Never married***** | 84(41.8) | 54(21.3) | 138(30.3) |
| Married or cohabiting***** | 112(55.7) | 178(70.1) | 290(64.0) |
| Divorced***** | 4(2.0) | 21(8.3) | 25(5.5) |
| Widowed | 1(0.5) | 1(0.4) | 2(0.4) |
| **Education history** |  | | |
| No education***** | 30(14.9) | 89(35.0) | 119(26.0) |
| Formal education***** | 171(85.1) | 165(65.0) | 336(73.9) |
| *Incomplete primary* | *59(29.4)* | *41(16.1)* | *100(22.0)* |
| *Primary* | *89(44.2)* | *101(39.8)* | *190(41.8)* |
| *Secondary/Post Secondary* | *23(11.4)* | *23(9.1)* | *46(10.1)* |
| **Occupation*** |  | | |
| Student | 38(18.9) | 25(9.8) | 63(18.9) |
| Farmer | 83(41.3) | 166(65.4) | 249(54.7) |
| Employed (government/private) | 62(30.9) | 49(19.3) | 111(24.4) |
| Unemployed | 18(9.0) | 14(5.5) | 32(7.0) |
