# Supplementary material for: Antiretroviral Treatment Knowledge and Stigma—Implications for Programs and HIV Treatment Interventions in Rural Tanzanian Populations
Source: PLoS One. 2013 Jan 16;8(1):e53993. doi: 10.1371/journal.pone.0053993 (PMC3546967; doi:10.1371/journal.pone.0053993)
Supplement: Table S3 — Characterizations of ART awareness, ART knowledge, ART-related knowledge and HIV-related stigma between men and women participating in the study. The answers provided in this table are positive answers. *It describes 694 participants of the 1000 individuals targeted in the survey. **Total sample = 455 (all respondents with non-missing ID or gender data that have heard of ART). Sample sizes for individuals vary somewhat due to different response pattern on each item. §At time of the study, ART was only accessible in two hospitals in the district. (DOC) [file pone.0053993.s003.doc]

**Table S3. Characterizations of ART awareness, ART knowledge, ART-related knowledge and HIV-related stigma between men and women participating in the study.**

**The answers provided in this table are positive answers. *It describes 694 participants of the 1000 individuals targeted in the survey. **Total sample=455 (all respondents with non-missing ID or gender data that have heard of ART). Sample sizes for individuals vary somewhat due to different response pattern on each item. ^§^At time of the study, ART was only accessible in two hospitals in the district.**

|  | **Gender** | | **P-value** | |  |
| --- | --- | --- | --- | --- | --- |
|  | Men (%) | Women (%) |  | Total (%) | |
| **ART community awareness** | | | | | |
| Have you ever heard about ART?* | 65.7 | 65.5 |  | 65.6 | |
| Where have you heard about ART?** |  |  |  |  | |
| *TV/Radio* | 62.2 | 49.2 | 0.006 | 54.9 | |
| *Poster* | 24.4 | 15 | 0.011 | 19.1 | |
| *Friends/family* | 38.8 | 40.1 |  | 39.6 | |
| *From health worker* | 28.9 | 48.1 | 0.001 | 39.6 | |
| **ART knowledge** | | | | | |
| ART is provided free of charge from hospital^§^ | 71.1 | 65.5 |  | 69.7 | |
| An HIV-infected pregnant woman can be on ART | 30.3 | 36.6 |  | 34.9 | |
| ART prolongs life for HIV-positive people | 19.4 | 24.6 |  | 21.3 | |
| ART is to be used life-long | 44.3 | 49.2 |  | 47 | |
| ART is to be used only when a person is very ill | 8.5 | 7.1 |  | 7.7 | |
| **ART-related stigma** | | | | | |
| ART patients are a threat to society | 58.7 | 53.5 |  | 55.8 | |
| ART patients are intentionally transmitting HIV | 54.3 | 44.1 | 0.05 | 44.6 | |
| ART patients look healthy after taking ARVs and transmit HIV to others | 42.8 | 33.9 | 0.03 | 37.8 | |
| ARVs increase sexual drive for ART patients | 44.8 | 47.2 | 0.01 | 46.1 | |
| ART patients are *"Dead to be"* | 36.8 | 38.6 |  | 37.8 | |
| ART patients will die soon | 10.9 | 9.8 |  | 10.3 | |
| **HIV-related stigma** | | | | | |
| People with HIV/AIDS are a threat to society | 63.2 | 62.2 |  | 62.6 | |
| People with HIV/AIDS are dangerous to me | 59.9 | 50.8 |  | 55.6 | |
| People with HIV/AIDS should be isolated from others | 51.3 | 56.3 |  | 54.5 | |
| People with HIV/AIDS are disgusting | 14.4 | 11.02 |  | 12.3 | |
| I do not want to be friend with people living with HIV/AIDS | 10 | 11.4 |  | 10.9 | |
