# Supplementary material for: Antiretroviral Treatment Knowledge and Stigma—Implications for Programs and HIV Treatment Interventions in Rural Tanzanian Populations
Source: PLoS One. 2013 Jan 16;8(1):e53993. doi: 10.1371/journal.pone.0053993 (PMC3546967; doi:10.1371/journal.pone.0053993)
Supplement: Table S4 — Fit indices for the LCA of ART knowledge, HIV-related stigma and ART-related stigma. Best fitting model is in bold. LCA = latent class analysis; AIC = Aikike Information Criterion; BIC = Bayesian Information Criterion; Adjusted BIC = Bayesian Information Criterion using sample size adjustment. *Number of latent classes restricted to education as the main grouping variable to make equal various conditional response probabilities across pairs of latent classes. (DOC) [file pone.0053993.s004.doc]

**Table S4. Fit indices for the LCA of ART knowledge, HIV-related stigma and ART-related stigma**

Best fitting model is in bold. LCA=latent class analysis; AIC= Aikike Information Criterion; BIC=Bayesian Information Criterion; Adjusted BIC= Bayesian Information Criterion using sample size adjustment. *Number of latent classes restricted to education as the main grouping variable to make equal various conditional response probabilities across pairs of latent classes.

|  | ART knowledge LCA models | | | | HIV-related stigma LCA models | | | | ART-related stigma LCA models | | | |
| --- | --- | --- | --- | --- | --- | --- | --- | --- | --- | --- | --- | --- |
| Description* | AIC | BIC | Adjusted BIC | Entropy | AIC | BIC | Adjusted BIC | Entropy | AIC | BIC | Adjusted BIC | Entropy |
| Two class, restricted | 55.68 | 101 | 66.1 | 0.58 | **65.55** | **110.87** | **75.96** | **0.83** | 132.75 | 186.32 | 145.06 | 0.7 |
| Three class, restricted | **48.94** | **119** | **65** | **0.63** | 56.09 | 126.13 | 72.18 | 0.82 | **106.8** | **189.2** | **125.73** | **0.63** |
| Four class, restricted | 51.4 | 146.2 | 73.2 | 0.71 | 58.46 | 153.22 | 80.23 | 0.76 | 94.36 | 205.61 | 119.92 | 0.67 |
| Five class, restricted |  |  |  |  | 67.36 | 186.85 | 94.81 | 0.61 | 97.84 | 237.94 | 130.03 | 0.71 |
