# Supplementary material for: Antiretroviral Treatment Knowledge and Stigma—Implications for Programs and HIV Treatment Interventions in Rural Tanzanian Populations
Source: PLoS One. 2013 Jan 16;8(1):e53993. doi: 10.1371/journal.pone.0053993 (PMC3546967; doi:10.1371/journal.pone.0053993)
Supplement: Table S5 — The latent class entities for ART knowledge and ART-related stigma. *Estimated conditional probabilities and membership probabilities (proportions) for each class based on “Yes” responses to the items. Bold numbers denote the most recurrent answer within the class to each statement. (DOC) [file pone.0053993.s005.doc]

Table S5. The latent class entities for ART knowledge and ART-related stigma

*Estimated conditional probabilities and membership probabilities (proportions) for each class based on "Yes" responses to the items. Bold numbers denote the most recurrent answer within the class to each statement.

| **Three latent class model on ART knowledge (n=455)** | | | |
| --- | --- | --- | --- |
|  | **Least informed** | **Moderately informed** | **Highly informed** |
| Membership probability | 0.24 | 0.08 | 0.68 |
|  | *Conditional probability of knowledge response** | | |
| ART is provided free of charge from hospital | **0.81** | **0.99** | **1.00** |
| An HIV-infected pregnant woman can be on ART | 0.18 | 0.37 | 0.54 |
| ART prolongs life for HIV-positive people | 0.37 | **0.60** | **0.95** |
| ART is to be used life-long | 0.04 | 0.32 | **0.71** |
| ART is to be used only when a person is very ill | **0.97** | 0.39 | 0.05 |
|  | | | |
| **Three latent class model on stigmatizing attitudes towards ART patients (n=455)** | | | |
|  | **Least stigmatizing** | **Moderately stigmatizing** | **Highly stigmatizing** |
| Membership probability | 0.28 | 0.41 | 0.31 |
|  | *Conditional probability of stigmatizing response** | | |
| ART patients are a threat to society | 0.19 | **0.76** | **0.81** |
| ART patients are intentionally transmitting HIV | 0.07 | 0.49 | **0.89** |
| ART patients look healthy after taking ARVs and transmit HIV to others | 0.10 | 0.44 | **0.99** |
| ARVs increase sexual drive for ART patients | 0.07 | 0.43 | **0.78** |
| ART patients are *"Dead to be"* | **0.76** | **0.99** | **0.94** |
| ART will die soon | 0.30 | **0.59** | **0.99** |
