# Supplementary material for: Antiretroviral Treatment Knowledge and Stigma—Implications for Programs and HIV Treatment Interventions in Rural Tanzanian Populations
Source: PLoS One. 2013 Jan 16;8(1):e53993. doi: 10.1371/journal.pone.0053993 (PMC3546967; doi:10.1371/journal.pone.0053993)
Supplement: Table S6 — Multinomial logistic regression models on ART knowledge, HIV-related stigma and ART-related stigma latent groups. Description on significant covariates at p<0.0001. The models were adjusted for; age, education, gender, marital status and occupation. Reference covariate and classes: *No formal education, †Least informed, **Low HIV-related stigma and § Least stigmatizing. Adjusted odds ratio and 95% confidence intervals. (DOC) [file pone.0053993.s006.doc]

**Table S6. Multinomial logistic regression models on ART knowledge, HIV-related stigma and ART-related stigma latent groups.**

Description on significant covariates at p<0.0001. The models were adjusted for; age, education, gender, marital status and occupation. Reference covariate and classes: *No formal education, ^†^Least informed, **Low HIV-related stigma and ^§^ Least stigmatizing. Adjusted odds ratio and 95% confidence intervals.

| ***Covariates*** | **Model 1** | | **Model 2** | **Model 3** | |
| --- | --- | --- | --- | --- | --- |
|  | *ART knowledge classes^†^* | | *HIV-related stigma classes*** | *ART-related stigma classes^§^* | |
|  | Moderately informed | Highly informed | Highly HIV-related stigma | Moderately stigmatizing | Highly stigmatizing |
| Education* |  |  |  |  |  |
| Primary education and above | 3.61(0.96-13.55) | 3.09(1.61-5.94) | 0.58(0.35-0.95) | 1.19(0.40-3.61) | 0.80(0.35-1.83) |
| *ART knowledge classes^†^* |  |  |  |  |  |
| Moderately informed | - | - | 0.35(0.13-0.92) | 1.13(0.18-7.29) | 0.83(0.19-3.58) |
| Highly informed | - | - | 0.44(0.24-0.81) | 0.29(0.08-1.10) | 0.26(0.09-0.74) |
| *HIV-related stigma classes*** |  |  |  |  |  |
| High HIV-related stigma |  |  | - | 42.15(43.43-57.43) | 61.57(55.40-72.50) |
| *Notes:* p<0.001. Only significant covariates at p<0.0001 are described in the table. Socio-demographic variables adjusted for in the model are; age, education, gender, marital status and occupation. Reference classes and covariates: *No formal education, ^†^Least informed, **Low HIV-related stigma and ^§^Least stigmatizing | | | | | |
